# Mixture of clopidogrel bisulfate and magnesium oxide tablets reduces clopidogrel dose administered through a feeding tube

**DOI:** 10.1186/s40780-021-00202-1

**Published:** 2021-05-04

**Authors:** Manahito Aoki, Midori Naya, Shiho Arima, Kaori Shinohara, Masahiro Kato, Kiyoshi Shibuya, Masaki Ohtawa, Tohru Nagamitsu, Katsuya Otori

**Affiliations:** 1 Department of Clinical Pharmacy, Research and Education Center for Clinical Pharmacy, School of Pharmacy, Kitasato University, 5-9-1 Shirokane, Minato-ku, Tokyo, 108-8641 Japan; 2 Department of Pharmacy, Kitasato University Medical Center, 6-100 Arai, Kitamoto, Saitama, 364-8501 Japan; 3 Department of Synthetic Natural Products Chemistry, Medicinal Research Laboratories, School of Pharmacy, Kitasato University, 5-9-1 Shirokane, Minato-ku, Tokyo, 108-8641 Japan

**Keywords:** Simple suspension method, Feeding tube, Magnesium oxide, Clopidogrel bisulfate

## Abstract

**Background:**

In clinical practice, a mixed suspension of clopidogrel bisulfate and magnesium oxide (MgO) tablets is administered frequently via a feeding tube. However, there is no report on the changes occurring when suspensions of these two drugs are combined, including the effects or potential decrease in dose following tube administration. Thus, the purpose of our study was to investigate the (i) changes caused by mixing clopidogrel bisulfate (ion form) and MgO tablets and (ii) effects on the administered clopidogrel dose after passing through a feeding tube.

**Methods:**

The molecular structure of clopidogrel generated in a mixture of clopidogrel bisulfate and a basic compound, such as sodium bicarbonate or MgO tablet, was determined by ^1^H-NMR after extraction and purification. The suspension of clopidogrel bisulfate tablet alone and the mixed suspension of clopidogrel bisulfate tablet and MgO tablet were passed through a feeding tube. We compared the yield of the molecular form of clopidogrel from each passed fraction.

**Results:**

The substance obtained from the mixture of clopidogrel bisulfate tablet and sodium bicarbonate or MgO tablet was identified as the molecular form of clopidogrel, and chemical degradation did not occur under these conditions. In the tube passage test, the yield of clopidogrel (molecular form) from the mixture of clopidogrel bisulfate and MgO tablets was lower than that from the suspension of clopidogrel bisulfate tablet alone.

**Conclusions:**

The mixture of clopidogrel bisulfate and MgO tablets caused a considerable reduction in the administered dose passed through the feeding tube. Therefore, it is recommended to administer the suspensions of clopidogrel bisulfate and MgO tablets separately for safe and effective pharmacotherapy.

## Background

A simple suspension method is widely used to administer drugs via a feeding tube; for patients who have difficulty swallowing, tablets or capsules may be disintegrated and suspended in hot water (55 °C) without crushing [1–3]. In clinical practice, nurses often simultaneously administer multiple drugs using this method. When two or more drugs are suspended together in the same syringe or cup, there is a risk of interactions or potential incompatibilities between them [4].

**Fig. 1:**
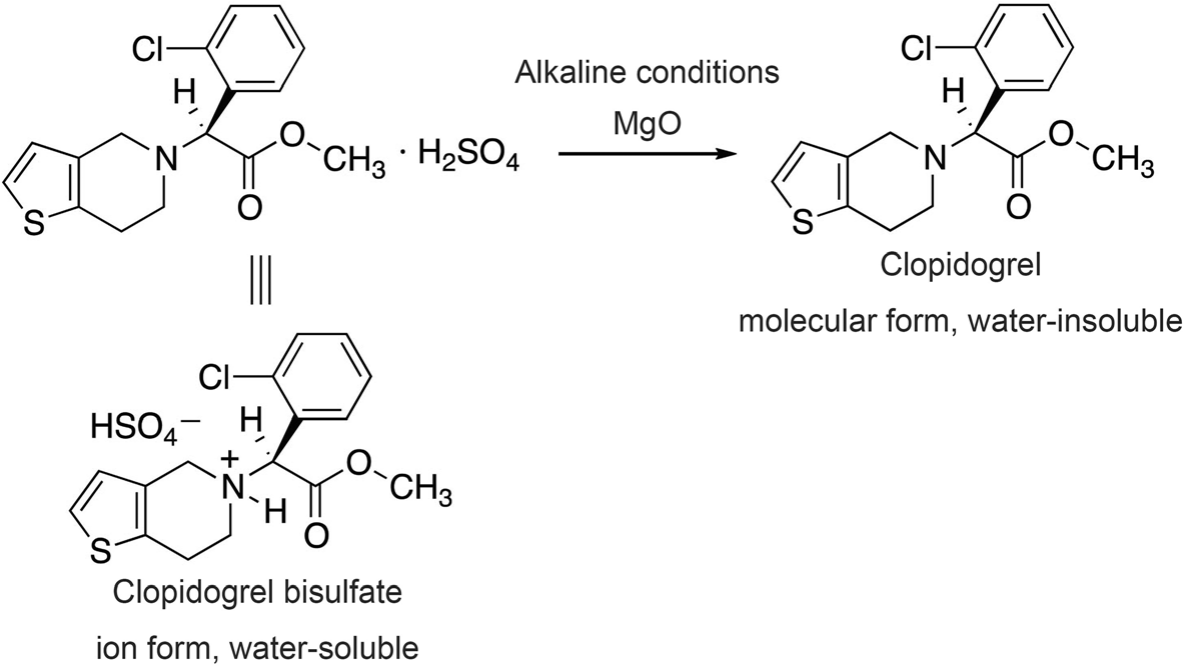
Chemical reaction of clopidogrel bisulfate under alkaline conditions

Previous studies have reported the stability of drugs by examining the changes following simultaneous suspension with alkaline drugs, such as magnesium oxide (MgO), or under pH-altered conditions [4–8]. Therefore, when multiple drugs are suspended together, attention should be paid to the suspension pH and changes after mixing. Pharmacists should review the prescription and propose a change if combining the prescribed drugs has the potential to cause changes. However, information on suspension pH and potential drug incompatibilities following the mixing of all drugs is limited. In clinical practice, clopidogrel bisulfate and MgO tablets are frequently prescribed together. The suspension of clopidogrel bisulfate tablets (Plavix^Ⓡ^, 75 mg) is acidic (average pH 2.3), whereas the MgO tablet (Magmitt^Ⓡ^, 330 mg) suspension is alkaline (average pH 10.5) [3]. Therefore, the mixed suspension of clopidogrel bisulfate and MgO tablets may cause chemical degradation and reduce the dose or change the water-soluble ionic form of clopidogrel to the water-insoluble molecular form, as shown in Fig. 1, and potentially create problems following tube administration. However, there is no report on such changes and problems. Therefore, we comprehensively investigated the potential changes that could occur following the cosuspension of clopidogrel bisulfate and MgO tablets.

## Methods

### Isolation and identification of the molecular form of clopidogrel obtained from the mixed suspension of clopidogrel bisulfate and a basic substance

#### Use of sodium hydrogen carbonate as a basic substance

**Fig. 2:**
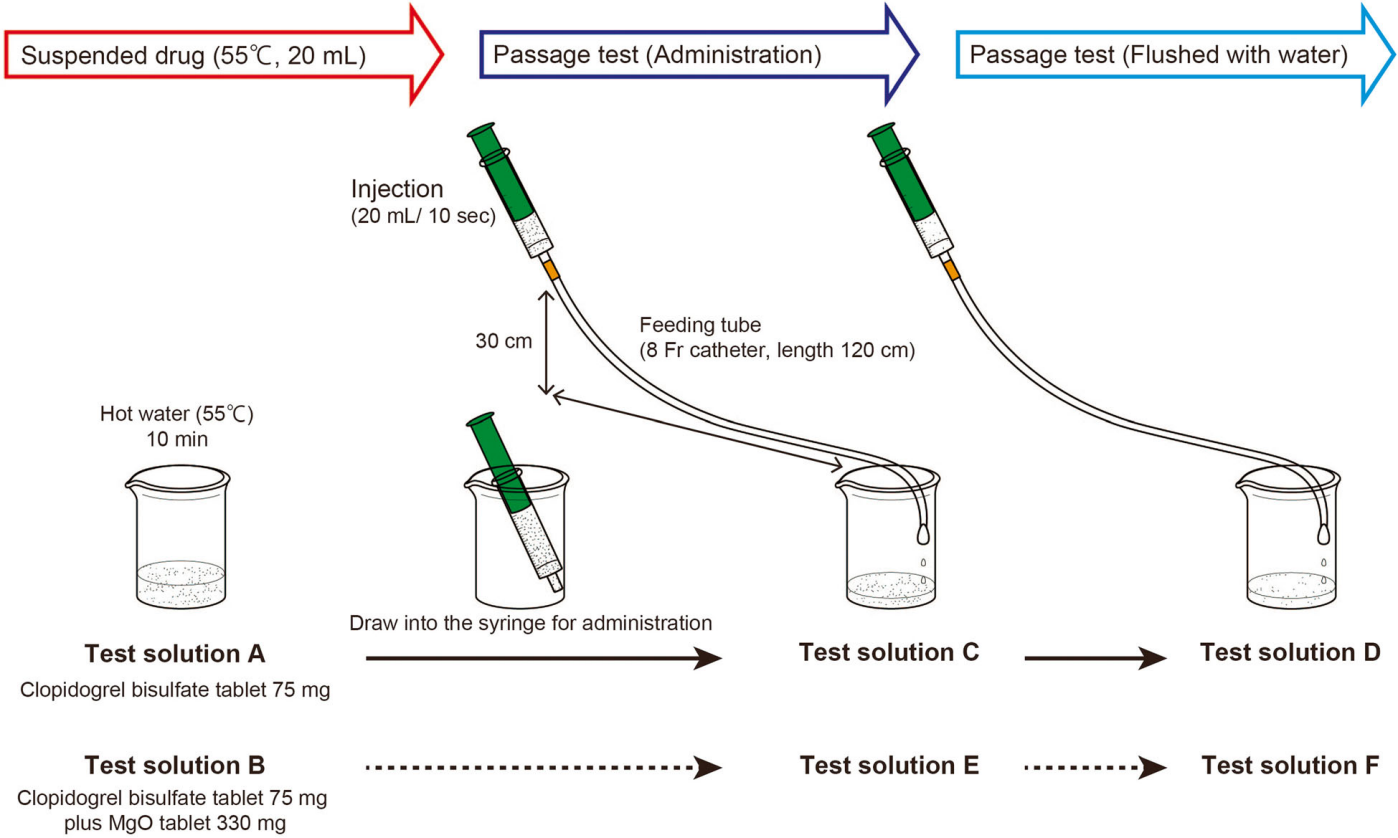
Test solution preparation and passage test

**Fig. 3:**
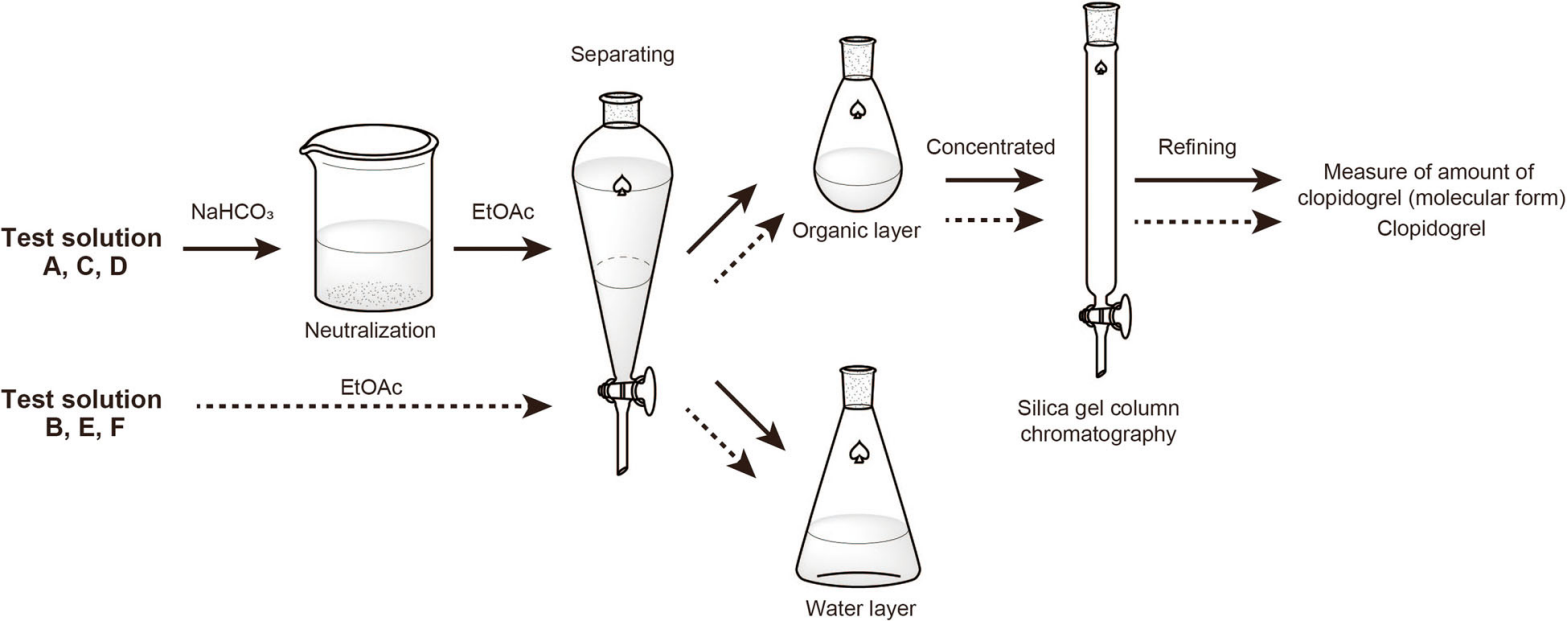
Process of extracting and purifying clopidogrel (molecular form) from test solutions. NaHCO_3_, sodium bicarbonate; EtOAc, ethyl acetate

A clopidogrel bisulfate tablet (Plavix^Ⓡ^, 75 mg, Japanese Pharmacopoeia clopidogrel bisulfate content, 97.88 mg/tablet; Sanofi, Tokyo, Japan) was crushed using a pestle, and then 20 mL of hot water (55 °C) was added. The mixture was allowed to stand for 10 min and was used as test solution A (Fig. 2). To identify whether the substance isolated by adding a basic substance to clopidogrel bisulfate suspension is clopidogrel (molecular form), test solution A was alkalified by adding a saturated aqueous solution of sodium hydrogen carbonate (NaHCO_3_). Using a pH test paper, it was confirmed that the test solution was weakly basic. The resulting mixture was extracted with ethyl acetate (EtOAc). The organic layer was washed with water and dried over anhydrous sodium sulfate. After filtering the mixture, the filtrate was concentrated under reduced pressure, the residue was purified by neutral flash silica gel column chromatography (50:1 hexane/EtOAc), and the dry weight of the resulting clopidogrel (molecular form) (Fig. 3) was measured and analyzed by ^1^H-NMR spectroscopy (the ^1^H-NMR spectrum of clopidogrel (molecular form) was obtained from test solution A).

#### Use of MgO tablets as a basic substance

Clopidogrel bisulfate tablet, crushed using a pestle, and MgO tablet (Magmitt^Ⓡ^, 330 mg, Japanese Pharmacopoeia MgO content, 330 mg/tablet, Kyowa Chemical Industry, Kagawa, Japan) were added to 20 mL of hot water (55 °C). The mixture was allowed to stand for 10 min and was used as test solution B (Fig. 2). Test solution B was extracted with EtOAc. The organic layer was washed with water and dried over anhydrous sodium sulfate. After filtering the solution, the filtrate was concentrated under reduced pressure, the residue was purified by neutral flash silica gel column chromatography (50:1, hexane/EtOAc), and the dry weight of the resulting clopidogrel (molecular form) (Fig. 3) was measured and analyzed by ^1^H-NMR spectroscopy (the ^1^H-NMR spectrum of clopidogrel (molecular form) was obtained from test solution B).

#### Comparison of substances obtained from test solutions A and B

The ^1^H-NMR spectra of clopidogrel (molecular form) obtained from test solutions A and B were compared with the spectra of an authentic sample (molecular form of clopidogrel) [9]. The yield of clopidogrel (molecular form) obtained from test solutions A and B is shown as mean ± standard deviation (*n* = 3).

### Determination of the estimated dose of clopidogrel (molecular form) administered using the clopidogrel bisulfate tablet or the mixed suspension of clopidogrel bisulfate and MgO tablets

#### Passage test using a feeding tube

Each test solution was subjected to a passage test, as shown in Fig. 2. Test solution A was drawn using a syringe (20-mL catheter tip syringe; SS-20CA20P, Terumo, Tokyo, Japan) and passed through a feeding tube (8 Fr catheter, external diameter 2.7 mm, external diameter of weighted part 4.6 mm, length 120 cm; Kangaroo™ New Enteral Feeding Tube; Covidien Japan, Tokyo, Japan), and the passed suspension was collected and used as test solution C. The syringe was filled with 20 mL of water, reconnected to the feeding tube, which was then flushed with water, and the passed suspension was collected and used as test solution D. The passage test [1] using test solution B was performed according to the procedure followed for test solution A, and the first passed suspension was used as test solution E, whereas the second passed suspension was used as test solution F. In the passage test, it is repeatedly evaluated whether a suspension passes through the tube and whether there is syringe plunger resistance during infusion compared with the syringe plunger resistance when only hot water (55 °C) is used during infusion. The evaluation was subjectively and qualitatively performed by three different experimenters.

#### Extraction and purification of clopidogrel (molecular form)

Isolation of clopidogrel bisulfate (ion form) from a tablet is more difficult than that of clopidogrel (molecular form). To isolate clopidogrel (molecular form) from test solutions C and D, which consisted of clopidogrel bisulfate (ion form), the solutions were alkalified by adding a saturated aqueous solution of NaHCO_3_; test solutions C, D, E, and F were extracted using EtOAc and each organic layer was washed with water and dried over anhydrous sodium sulfate. After filtration, each filtrate was concentrated under reduced pressure and the residues were purified by neutral flash silica gel column chromatography (50:1 hexane/EtOAc), and the dry weight of the resulting clopidogrel (molecular form) (Fig. 3) was measured.

#### Evaluation of the estimated dose of clopidogrel (molecular form)

The Japanese Pharmacopoeia 17^th^ edition stipulates that the quantitative analysis of clopidogrel bisulfate tablets should confirm whether it contains 95.0–105.0% of the labeled amount of clopidogrel [10]. Accordingly, we verified whether the estimated administered dose was within the stipulated range (71.25–78.75 mg) and compared the effect of suspending the tablets on the dose.

• Estimated dose administered via a feeding tube using a suspension of only clopidogrel bisulfate tablets = total amount of clopidogrel (molecular form) recovered from test solutions C and D.

• Estimated dose administered via a feeding tube using the mixed suspension of clopidogrel bisulfate and MgO tablets = total amount of clopidogrel (molecular form) recovered from test solutions E and F.

### Measurement of the pH of suspensions

We measured the pH of suspensions of clopidogrel bisulfate tablet alone, MgO tablet alone, and the mixed suspension of both. The suspensions were prepared by adding one tablet of each drug to 20 mL of hot water (55 °C) in a syringe, attaching a cap (ED-CP cap for catheter tip syringe, Terumo, Tokyo, Japan), and inverting the syringe 15 times to agitate the contents before measuring the pH using a pH meter (FiveGo™ FG2; Mettler Toledo, Switzerland). The observed pH values of suspensions are shown as mean ± standard deviation (*n* = 3).

### Observation of the appearance of suspensions of clopidogrel bisulfate tablet alone, MgO tablet alone, and the mixed suspension of both during and after the passage test

At each stage, we observed the appearance of the suspension of clopidogrel bisulfate tablet alone, MgO tablet alone, and the mixed suspension of both. After the passage test of each suspension, we confirmed residual substance in the syringe, gasket, tube, and tube connection site. Photographs of each suspension in the syringe were taken and visual changes in the appearance were recorded. We touched the residue in the syringe and gasket surface to confirm the status.

### Statistical analysis

The estimated dose of clopidogrel (molecular form) in the single-agent clopidogrel bisulfate suspension was compared with that in the mixed suspension of clopidogrel bisulfate and MgO tablets using an unpaired *t*-test with EZR [11].

## Results

### Confirmation of the yield and structure of clopidogrel (molecular form) obtained from test solutions A and B

**Fig. 4:**
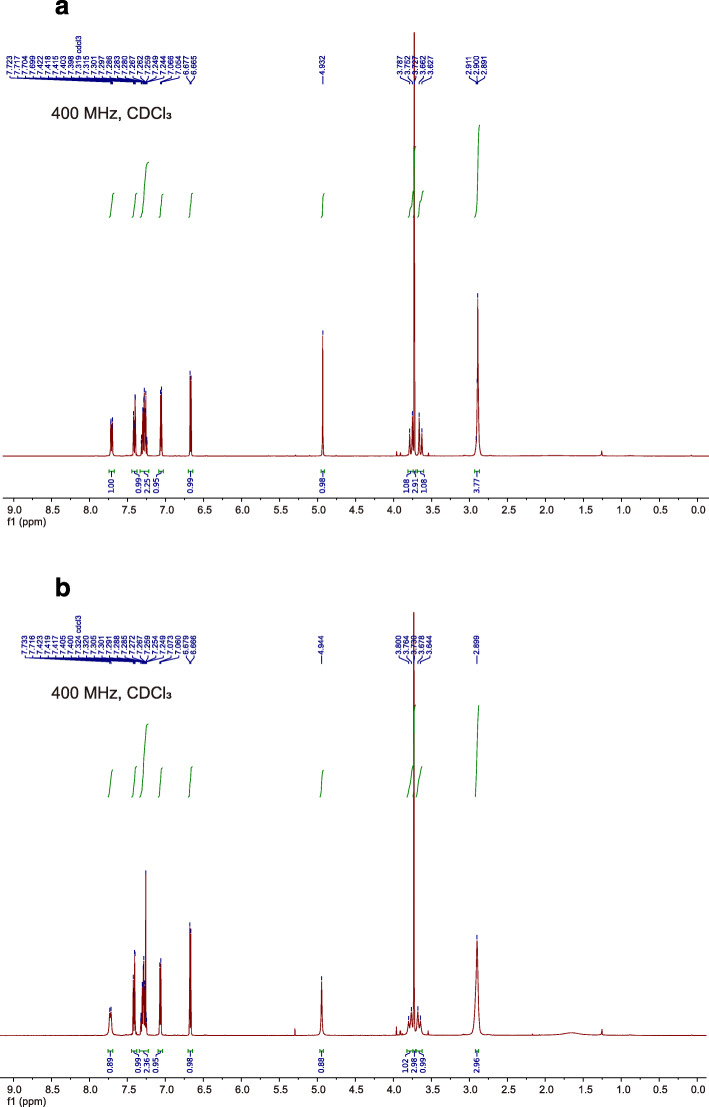
Comparison of the ^1^H-NMR spectra of substances obtained from test solutions **a** and **b**. **a** and **b** ^1^H-NMR spectral findings for clopidogrel (molecular form) obtained from test solutions **a** and **b**, respectively

The ^1^H-NMR spectra of clopidogrel (molecular form) obtained from test solutions A and B matched those previously reported for clopidogrel (molecular form), as shown in Fig. 4A and B [9]. The yield of clopidogrel (molecular form) obtained from test solutions A and B was 74.0 ± 0.3 (*n* = 3) and 74.7 ± 0.9 mg (n = 3), respectively. The desired clopidogrel (molecular form) was quantitatively recovered from test solutions A and B. This result indicates that chemical degradation does not occur by mixing clopidogrel bisulfate and MgO tablets.

### Yield of clopidogrel (molecular form) after the passage test

**Fig. 5:**
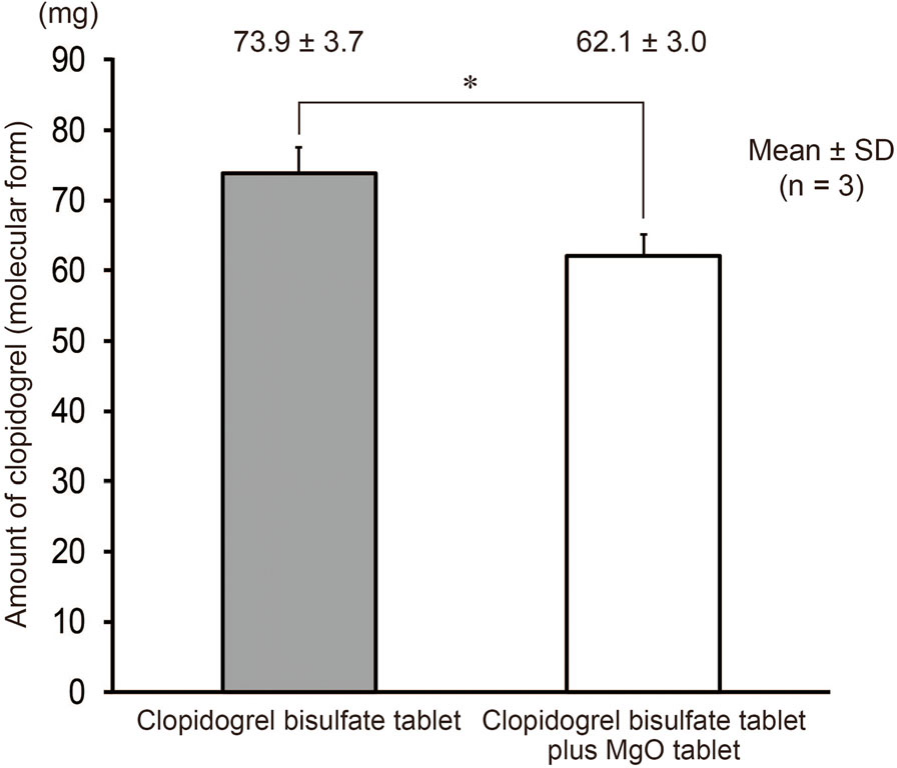
Estimated dose of clopidogrel (molecular form). In the passage test using the cosuspension of clopidogrel bisulfate and MgO tablets, tube blockage occurred in two of the five tests. Therefore, data of the two tests were not included in this analysis. * *p* < 0.05

There was no blockage during the tube passage test using test solution A. The yield of clopidogrel (molecular form) recovered from test solutions C and D (using a suspension of clopidogrel bisulfate tablets alone) was 73.9 ± 3.7 mg (*n* = 3), which was within the stipulated range, whereas that from test solutions E and F (using a cosuspension of clopidogrel bisulfate and MgO tablets) was lower (62.1 ± 3.0 mg, n = 3) than the stipulated range (Fig. 5). With test solution B, tube blockage occurred in two of the five tests. Therefore, the data of the two tests were not included in this analysis.

### pH values and appearance of suspensions of the clopidogrel bisulfate tablet alone, MgO tablet alone, and the mixed suspension of both

**Fig. 6:**
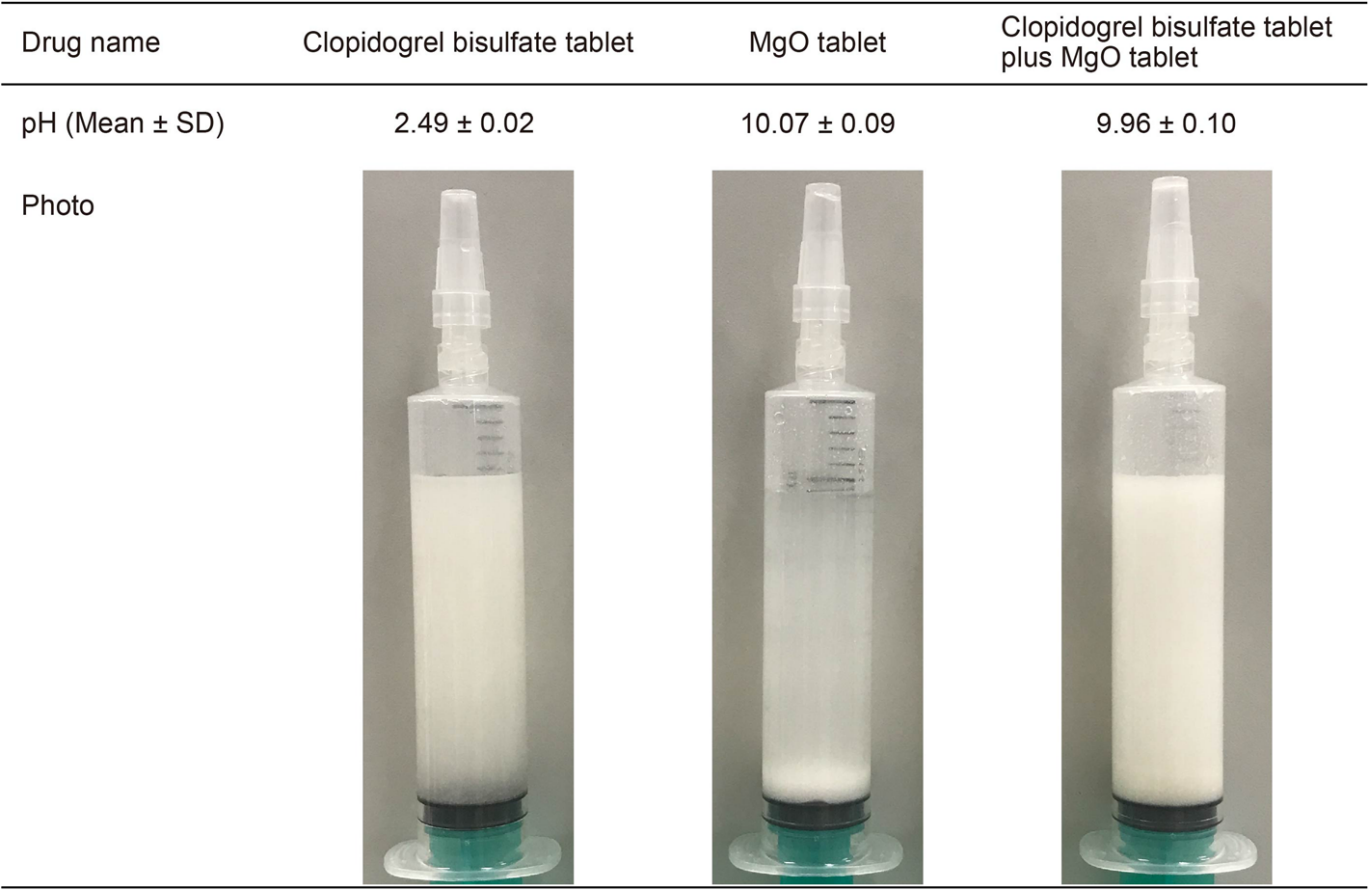
Changes in clopidogrel bisulfate and MgO tablet single suspensions and cosuspensions in syringes and pH

The pH of the suspensions of clopidogrel bisulfate tablet alone and MgO tablet alone was 2.49 ± 0.02 and 10.07 ± 0.09, respectively, which were similar to those reported previously [3]. The pH of the mixed suspension of clopidogrel bisulfate and MgO tablets was 9.96 ± 0.10. It appeared that the addition of MgO tablet to clopidogrel bisulfate tablet increased the cloudiness of the suspension (Fig. 6).

### Results observed during and after the passage test

**Fig. 7:**
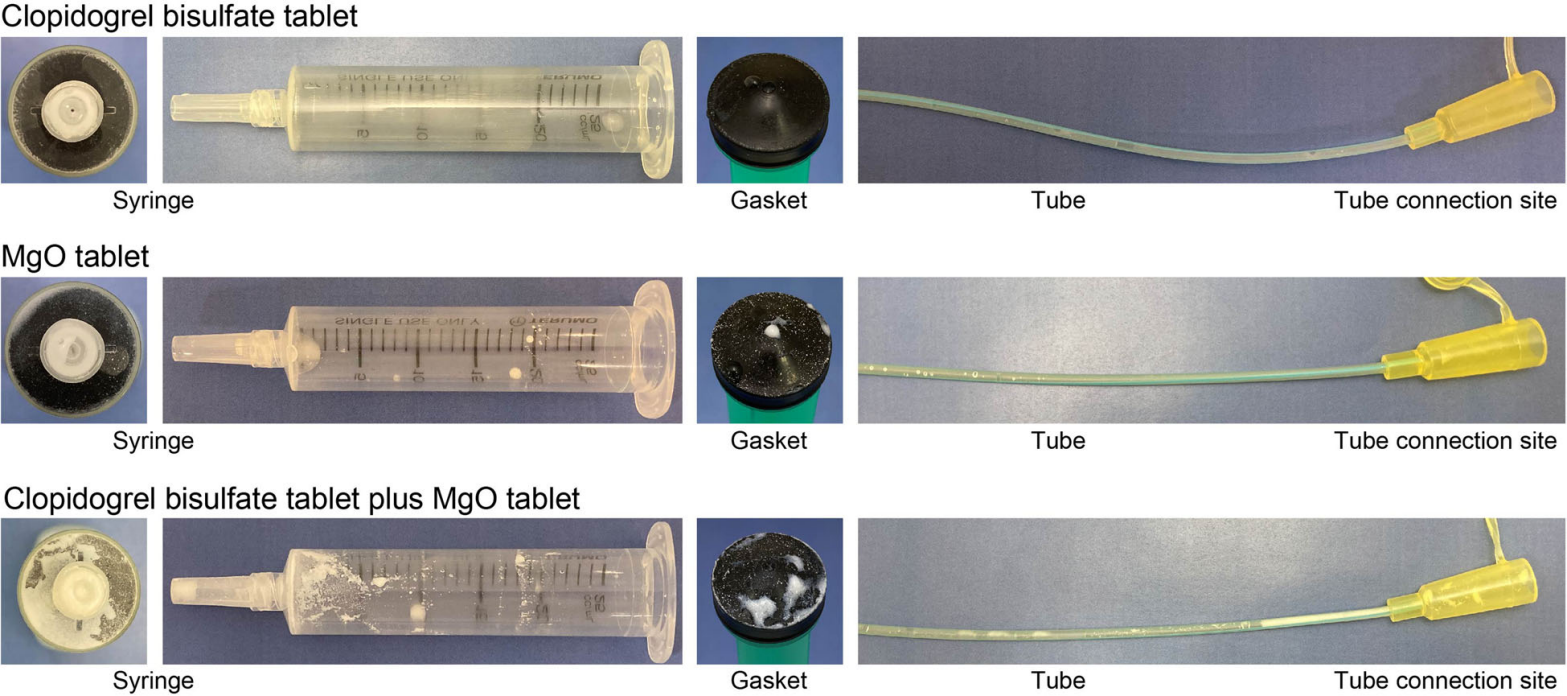
Comparison of the appearance of syringe, gasket, and tube connection site after the passage test

Based on the evaluation by all three experimenters, there was no change in the syringe plunger resistance when the clopidogrel bisulfate tablet suspension was passed through an 8-Fr tube during the passage test. In contrast, all experimenters observed that the mixed suspension of clopidogrel bisulfate tablet and MgO tablet showed plunger resistance and poor passage through the feeding tube. Moreover, in the mixed suspension of clopidogrel bisulfate tablet and MgO tablet, residual white and viscous substance was observed in the syringe, gasket, and tube connection site after the passage test, compared with the suspension of clopidogrel bisulfate tablet alone or MgO tablet alone (Fig. 7).

## Discussion

The ^1^H-NMR spectra of clopidogrel (molecular form) obtained from the mixed suspension of clopidogrel bisulfate tablet and the basic sodium hydrogen carbonate (test solution A) matched those obtained from the mixed suspension of clopidogrel bisulfate and the basic MgO (test solution B). This indicates that a neutralization reaction occurred between the acidic and basic substances in the suspension, as shown in Fig. 1, and it changed clopidogrel from the water-soluble ionic form to the water-insoluble molecular form.

It has been reported that cosuspending drugs such as aspirin [5], amlodipine besilate [6], and capecitabine [7] with MgO or other agents that increase the suspension pH reduces the amount of the principal agent in the suspension by degradation or other changes. With clopidogrel bisulfate, the amount of clopidogrel (molecular form) isolated from test solutions A and B was similar to the amount of clopidogrel bisulfate used to prepare the test solutions, and this indicates that formulating these test solutions caused no degradation. However, after the passage test, viscous white substance adhered to the inner side of the syringe, gasket, and tube connection site was observed (Fig. 7), and the resistance of the syringe plunger increased, which made it difficult to depress. Furthermore, our results of the passage test to determine whether clopidogrel (molecular form) remained in the syringe or feeding tube showed that the suspension of clopidogrel bisulfate tablet alone delivered almost the entire dose, whereas the mixed suspension delivered a considerably less dose (Fig. 5). The reduced dose of clopidogrel (molecular form) suggested that the water-insoluble substances including clopidogrel (molecular form), which was formed in the mixed suspension of clopidogrel bisulfate and MgO tablets, adhered to the inner side of the syringe or tube. We did not confirm that the viscous substance attached to the syringe plunger was clopidogrel (molecular form), and it cannot be denied that the residue may be another substance generated by the interaction with the additive of MgO tablet. However, clopidogrel (molecular form) is a viscous oily liquid [12, 13]. Therefore, this phenomenon can be attributed to the changes in the physical properties of clopidogrel (ion form) to water-insoluble and viscous clopidogrel (molecular form) via neutralization under alkaline conditions. This reduction in dose caused by mixing clopidogrel bisulfate and MgO tablets not only reduces the therapeutic effect in patients but is also economically wasteful as it amounts to approximately one less dose every five doses. To ensure effective and safe pharmacological treatment, the use of the mixed suspension of clopidogrel bisulfate and MgO tablets should be avoided and the suspension of each agent should be administered separately.

We have shown that the acidic suspension of clopidogrel bisulfate tablets causes a neutralizing reaction with the basic suspension of MgO or NaHCO_3_ to generate clopidogrel (molecular form) without degradation. The use of the mixed suspension of clopidogrel bisulfate tablet and MgO reduced the actual administered dose of clopidogrel. Moreover, with test solution B, tube blockage occurred in two of the five passage tests, suggesting that the cosuspension of clopidogrel bisulfate and MgO tablets may increase the risk of tube blockage. Hence, mixing these agents as a suspension should be avoided.

At Kitasato University Medical Center, pharmacists in the Department of Neurology/Department of Neurosurgery ward monitor the prescriptions of patients who receive tube feeding and provide nurses with relevant information. Nurses frequently seek advice on tube administration of drugs from pharmacists, who confirm whether the prescribed drugs can be safely administered through this route as suspensions. In addition, pharmacists provide information on how to prevent and avoid exposure to hazardous drugs [14], changes when mixing drugs, and other formulation-related matters. Based on the findings of this study, nurses have been informed to avoid changes caused by mixing acidic clopidogrel bisulfate and basic MgO tablets.

We determined the pH of drug suspensions based on existing information [3] and measurements and used this information in clinical practice. However, information available on the pH of drug suspensions is scarce. Determining the pH of suspensions, changes that occur when several agents are mixed, and the actual administered dose could increase the safety and therapeutic effect of drug administration via a feeding tube in clinical practice. We expect more reports on these safety-increasing evaluations of the simple suspension method, and hope that the findings will be implemented in clinical practice.

## Conclusions

The results of this study indicate that the administration of a mixed suspension of clopidogrel bisulfate and MgO tablets via a feeding tube reduces the actual dose and increases the risk of tube blockage because of the adherence of water-insoluble substances including clopidogrel (molecular form). Finally, for safe and effective pharmacotherapy, we emphasize that clopidogrel bisulfate tablets should not be mixed with MgO tablets or alkaline agents as a suspension in hot water; they should rather be suspended and administered separately.

## Data Availability

The dataset supporting the conclusions of this study is included within the article.

## Abbreviations

EtOAc: Ethyl acetate
MgO: Magnesium oxide
NaHCO_3_: Sodium hydrogen carbonate
